# NLRP3 genetic polymorphisms are associated with the onset of mild cognitive impairment in Chinese

**DOI:** 10.1002/alz.087252

**Published:** 2025-01-03

**Authors:** Ruonan Gao, Suk Ling Ma

**Affiliations:** ^1^ The Chinese University of Hong Kong, Hong Kong Hong Kong; ^2^ The Chinese University of Hong Kong, Hong Kong, Hong Kong Hong Kong

## Abstract

**Background:**

Nucleotide‐binding domain and leucine‐rich repeat (LRR)‐containing family protein 3 (NLRP3) is involved in neuroinflammation in Alzheimer’s Disease (AD). Single nucleotide polymorphisms (SNPs) in the NLRP3 gene are associated with the risk of AD in different populations, however the relationship between NLRP3 SNPs and Hong Kong population has not been studied.

**Method:**

In this study,12 intron SNPs and 2 exon SNPs were genotyped in 233 healthy controls and 323 mild cognitive impairments (MCI) patients from Hong Kong. Gene expression levels of NLRP3 and other inflammation‐related genes were evaluated in peripheral blood mononuclear cells (PBMC) from the subjects by quantitative PCR (qPCR).

**Result:**

Our results showed that rs10754558 (C/T) and rs7525979 (G/C) were associated with an increased risk of MCI. The T allele of rs12564791 was associated with higher gene expression level of NLRP3 (p<0.001), IL18 (p<0.001) and IL1beta (p = 0.044) in PBMC. The G allele of rs3806268 was negatively correlated with NLRP3 gene expression (p = 0.048). Functional studies by dual‐luciferase assay showed that rs10754558 G to C mutation and rs10754555 G to C mutation led to a lower relative luciferase activity.

**Conclusion:**

These results suggested that genetic variations of NLRP3 were associated with the cognitive decline. In addition, NLRP3 SNPs might be associated to the gene expression of NLRP3.

